# Delay in Diagnosis of Cerebral Venous and Sinus Thrombosis: Successful Use of Mechanical Thrombectomy and Thrombolysis

**DOI:** 10.1155/2011/815618

**Published:** 2011-07-09

**Authors:** Christopher T. Shah, Jason J. Rizqallah, Oladoyin Oluwole, Aleksandrs Kalnins, John N. Sheagren

**Affiliations:** ^1^College of Human Medicine, Michigan State University, Grand Rapids, MI 49503, USA; ^2^Internal Medicine Residency, Grand Rapids Medical Education Partners, Grand Rapids, MI 49503, USA; ^3^Radiology Residency, University of North Carolina at Chapel Hill, Chapel Hill, NC 27514-4423, USA; ^4^Department of Internal Medicine, Grand Rapids Medical Education Partners, Grand Rapids, MI 49503, USA

## Abstract

Cerebral venous and sinus thrombosis is a relatively rare condition with a variable presentation that can translate into a difficult workup and a delay in diagnosis and treatment. We describe the successful use of mechanical thrombectomy and thrombolysis in the case of an eighteen-year-old woman that presented with progressive thrombosis of the jugular veins and dural sinuses despite adequate anticoagulation. Our case highlights the need for clinicians to include CVST in the initial differential diagnosis of patients in order to prevent delays and poor outcomes.

## 1. Introduction

Cerebral venous and sinus thrombosis (CVST) is a relatively rare clinical condition affecting about 5 people per million [1] and complicated by a nonspecific, highly variable presentation that can lead to a significant delay in diagnosis [1, 2]. Misdiagnosis and delay in diagnosis are common, with an initial misdiagnosis occurring in up to 73% of patients and delays exceeding 10 days in 40% of patients [3]. In the Western world, the main causes of CVST are genetic or acquired prothrombotic factors, whereas infection predominates the clinical picture in developing countries [2, 4, 5]. As a result of gender-specific acquired risk factors, a female predominance among patients suffering CVST has been documented [2], with a ratio of 3 : 1 women to men in young to middle-aged adults [4]. Here we report a challenging diagnostic workup for a case of progressive thrombosis of the jugular veins and dural sinuses despite adequate anticoagulation, necessitating mechanical thrombectomy and thrombolysis. A successful outcome was achieved.

## 2. Case Report

An 18-year-old woman was transferred to our tertiary care hospital after presenting to a community Emergency Department (ED) with increased intraocular pressure (IOP), papilledema, accompanied by bilateral jugular vein thrombosis (JVT) and dural venous sinus thrombosis (DVST) confirmed by head CT scan. On arrival, the patient presented with blurred vision, more specifically progressive loss of vision on the left side upon closing right eye. Of note, the patient's mother had a history of diabetes mellitus, hypertension, and dyslipidemia and had experienced a possible CVA at age 33. The patient herself has a history significant for morbid obesity, polycystic ovarian syndrome, and insulin resistance. The patient denied any history of smoking, tobacco, drugs, or alcohol use, as well as any speech abnormalities, dysphagia, tingling, numbness, weakness, bowel or bladder incontinence. 

Two months prior to admission (PTA), the patient had started taking a third-generation oral contraceptive pill (OCP), norgestimate and ethinyl estradiol tablets, which was stopped 3 weeks later after she developed headaches. One month PTA, the patient developed an “extremely bad” headache, followed by nausea/vomiting, bilateral neck pain, and right ear pain prompting presentation to ED where antibiotics were prescribed for suspected acute otitis media. Two weeks later, the patient returned to the ED with similar symptoms and was subsequently diagnosed with bilateral mastoiditis; tympanostomy tubes were placed bilaterally. Symptoms persisted, however, and she subsequently developed blurred vision prompting her return to the ED for a third time, two days later. A head CT scan with contrast demonstrated left jugular venous thrombosis prompting hospital admission. The patient was placed on IV Coumadin, with a Lovenox bridge. She was subsequently discharged on Coumadin (5 mg PO daily) and Lovenox (100 mg subQ BID) and was referred to an ophthalmologist. Six days after hospital discharge, the ophthalmologist noted her to have increased IOP along with papilledema. She was emergently referred to the ED, where a head CT scan demonstrated extension of the thrombotic process to include the dural venous sinus and bilateral jugular veins. These findings prompted the patient's transfer to our care for further evaluation and treatment. 

**Figure 1:**
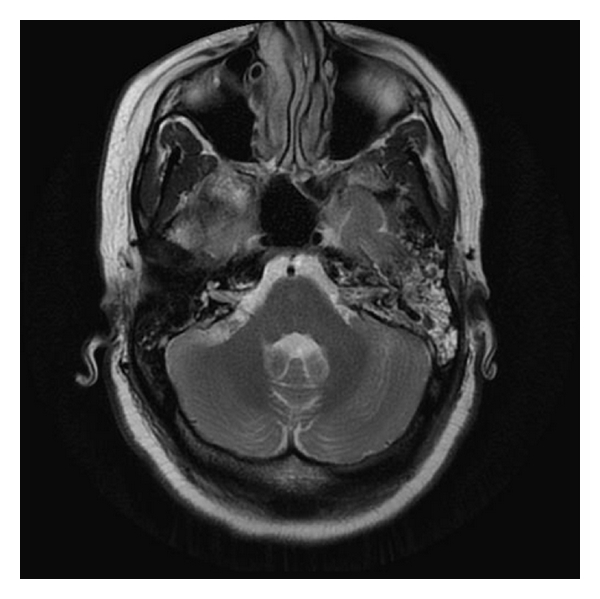
MRI of brain with contrast demonstrating fluid and enhancement within the mastoids, more extensive on the left, suggestive of mastoiditis.

**Figure 2:**
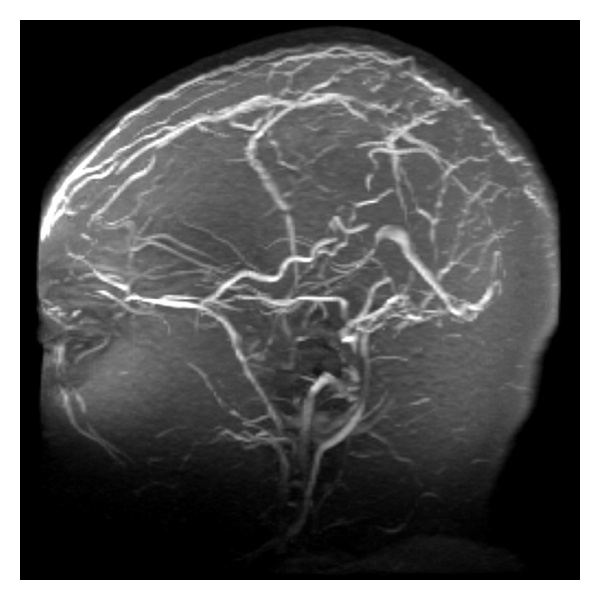
MRV of head without contrast demonstrating absence of normal flow-related enhancement within the sagittal, transverse, and left sigmoid sinus suggestive of dural sinus thrombosis. There are small adjacent dural vessels in proximity to these thrombosed sinuses.

Neurological evaluation demonstrated papilledema and markedly impaired left eye vision, with no other focal neurological deficits. Coagulation studies demonstrated supratherapeutic anticoagulation levels with PT 33.5, INR 3.6, and APTT 33. Blood work including CBC and electrolytes was normal. Cardiolipin screen and blood cultures (x2) were negative. A hypercoagulability work-up screening for antithrombin III levels, protein C and S, factor VIII, factor V Leiden, lupus anticoagulant, prothrombin G210A mutation, and homocysteine returned unremarkable. MRI of the brain with and without contrast revealed no evidence of an acute ischemic event but did show evidence of dural and cortical venous thrombosis and findings consistent with mastoiditis (see Figure 1). MR venography (MRV) of the head without contrast demonstrated DST with areas of apparent collateralization and recanalization (see Figure 2). 

Interventional radiology was consulted for mechanical thrombectomy and thrombolysis with tissue plasminogen activator (tPA), both of which were successfully performed. Ophthalmological evaluation after the procedure demonstrated no afferent papillary defect, clearing of papilledema, and decreased visual acuity bilaterally with left eye 20/100 and right eye 20/200. Follow-up CT cerebral angiography revealed improvement in the appearance of both transverse venous sinuses and the superior sagittal sinus when compared to previous images. The patient's vision continued to improve, and she was discharged home on Coumadin (5mg daily) with directions to follow up with an ophthalmologist. 

## 3. Discussion

In this case, the patient's nonspecific presentation resulted in a delay of two weeks from symptom onset to the diagnosis of left jugular vein thrombosis and offers a number of teaching points. A review of common presentations, etiology, workup, and treatment will follow to help clinicians effectively include CVST in their differential diagnosis and to prevent a delay in diagnosis which may result in poor outcomes.

The initial signs and symptoms of CVST can mimic more common disorders causing a delay in diagnosis [1]. The clinical picture typically falls under one of four distinct syndromes: (1) isolated intracranial hypertension, (2) focal neurological deficits, (3) encephalopathy, and (4) cavernous sinus syndrome [1, 2]. Each syndrome has its own unique clinical presentation: intracranial hypertension presents with headache with or without papilledema and visual disturbances, focal neurological deficits with hemi- or monoparesis, encephalopathy with an altered level of consciousness, and cavernous sinus syndrome with oculomotor nerve palsies [2]. Of the various presenting symptoms the most common is headache [2–6], as seen in our patient. The clinical picture is also dependent on the time elapsed from symptom onset to presentation for medical care, as patients with a chronic course or delayed presentation can show papilledema which is less common in acute cases [1].

In addition to a variable clinical presentation, the etiology of CVST is commonly multifactorial [1, 7], with a large portion of patients having more than one cause or predisposing factor [1, 3, 8]. Our patient's recent history of starting a third-generation OCP and its temporal relationship to the onset of headaches should have warranted that CVST at least be included in the differential diagnosis. Oral contraceptives are a well-known risk factor for CVST [2–5] with third-generation contraceptives substantially increasing the risk of venous thrombosis and CVST [2, 9]. In an International Study on Cerebral Vein and Dural Sinus Thrombosis, 54% of the 381 women younger than 50 years of age had oral contraceptive use as a risk factor [8]. In hindsight, mastoiditis was another clue to the diagnosis of CVST, due to the fact that mastoiditis is a known cause of lateral venous sinus thrombosis (LST) [10]. A retrospective review of otogenic LST at The Children's Hospital of Philadelphia found that such patients most commonly presented with headache and a history of treatment for acute otitis media (6–60 days). In about 60% of the cases, symptoms lasted >2 weeks [6]. Other risk factors include antithrombin deficiency, protein C and S deficiency, factor V Leiden mutation, hyperhomocysteinemia, nephrotic syndrome, antiphospholipid antibody syndrome, pregnancy, the puerperium, and meningitis [5]. 

As part of the workup of CVST, a hypercoagulable workup should be performed, preferably before the initiation of anticoagulation therapy, since heparin and warfarin can alter some lab results [11]. Although our patient had had an unremarkable hypercoagulable workup, she presented to us already on warfarin and will require further confirmatory labs after warfarin is stopped. In searching for an etiology, imaging is typically used. With our patient, a head CT scan was the initial imaging study; however, magnetic resonance imaging is the most sensitive tool for detecting CVST [2, 3] and subtraction angiography the gold standard [3]. The combination of MR angiography and venography is now the first imaging choice for diagnosing CVST [2, 6]. Depending on the time course of CVST, the MR imaging signal appearance is variable with isointense T1-weighted images and hypointense T2-weighted images during the first 3–5 days. After the first 5 days, the T1- and T2-weighted MR images are of high intensity, whereas one month after such images may become variable or appear isointense [2]. 

Current therapy is centered on identifying and managing any underlying predisposing factors; management of elevated intracranial pressure and cerebral edema; symptomatic treatment for seizures, headaches, and visual disturbance; achieving appropriate anticoagulation, even in the presence of intracranial hemorrhage [2, 3, 12]. Endovascular treatment of CVST is not currently included in the existing treatment guidelines and should probably be a last resort reserved for selected severe cases [2], as it was used in our patient. A number of case reports have described the use of urokinase or recombinant tPA in cases where anticoagulation was unsuccessful; however, the reports noted that such patients experienced higher rates of intracranial hemorrhages, especially when preexisting intracerebral hemorrhage was present [2]. Despite this concern, mechanical thrombectomy has shown promising results and still presents an attractive option even in patients with preexisting intracranial hemorrhage [3, 12–14]. In 2009, Rahman et al. [12] published a recommended treatment algorithm for CVST that used GCS cutoffs for systemic anticoagulation versus tPA infusion and/or thrombectomy. Until randomized clinical trials are undertaken, it must be understood that this procedure is still considered investigational.

In summary, our case highlights the need for clinicians to include CVST in the initial differential diagnosis of patients in order to prevent delays and poor outcomes. Such patients include young women with a history of recent initiation of an oral contraceptive, mastoiditis, altered vision, or neurological deficits. Physicians should have a lower threshold for ordering neuroimaging studies when faced with the scenario described above. In addition, we report the successful use of mechanical thrombectomy and thrombolysis with tPA in the face of failure of anticoagulation treatment.

## Abbreviations

CVST: Cerebral venous and sinus thrombosis
DVST: Dural venous sinus thrombosis
JVT: Jugular vein thrombosis
LST: Lateral venous sinus thrombosis
MRV: MR venography
PTA: Prior to admission
OCP: Oral contraceptive pill
tPA: Tissue plasminogen activator.
